# Effect of oral meloxicam administration on growth performance and behavior of pre-weaning age calves following band castration

**DOI:** 10.1093/tas/txaa021

**Published:** 2020-02-17

**Authors:** Jay A Daniel, Alison Crane, Peter D Krawczel, Johann F Coetzee, Brian K Whitlock

**Affiliations:** 1 Department of Animal Science, Berry College, Mt. Berry, GA; 2 Department of Animal Sciences and Industry, Kansas State University, Manhattan, KS; 3 Department of Animal Science, University of Tennessee, Knoxville, TN; 4 Department of Anatomy and Physiology, Kansas State University, Manhattan, KS; 5 Department of Large Animal Clinical Sciences, College of Veterinary Medicine, University of Tennessee, Knoxville, TN

**Keywords:** behavior, calf, castration

## Abstract

The objective of this study was to determine if oral meloxicam (M; a nonsteroidal anti-inflammatory drug) administered at castration to pre-weaning age calves affected average daily gain (ADG) or behavior. Prior to castration (d −14), Angus bulls were weighed and randomly assigned to be band castrated (BAN; *n* = 8; age = 90.2 ± 6.5 d; BW = 146.3 ± 11.4 kg; scrotal circumference = 16.0 ± 0.5 cm) or castrated with M (BAN + M; *n* = 9; age = 102.1 ± 6.2 d; BW = 146.0 ± 7.7 kg; scrotal circumference = 16.1 ± 0.3 cm). Six bulls selected to remain bulls based on pedigree and phenotype were maintained in the same pasture (BULL; age = 104.2 ± 6.1 d; BW = 172.1 ± 8.7 kg; scrotal circumference = 17.5 ± 0.4 cm). On d 0, BAN and BAN + M had a rubber band applied tightly around the scrotum, and BAN + M also received oral M (2 mg/kg BW). On d 1, 14, and 28, animals were weighed and a blood sample was collected to determine circulating concentrations of haptoglobin and fibrinogen. Data loggers were affixed to the legs of calves immediately prior to castration (d 0) to record behaviors [mean lying time (h/d), mean lying bouts (*n*/d), and steps (*n*/d)] at 1-min intervals and removed on d 28. Behavior and plasma data were tested for effect of treatment, day, and treatment × day interaction, and ADG data were tested for effect of treatment, period (d −14 to 1, d 1 to 14, and d 14 to 28), and treatment × period interaction using JMP procedures for repeated measures (SAS Inst. Inc., Cary, NC). BULL in period d 0 to 14 had greater ADG than all other treatment period combinations, and BULL had greater ADG than BAN or BAN + M overall (*P* < 0.05). There was no effect of M treatment on circulating concentrations of fibrinogen or haptoglobin (*P* > 0.05). On d 7 and 15, BAN took more steps than BAN + M (*P* < 0.05). BAN + M had more lying bouts than BAN on d 13 and 14 (*P* < 0.05). Overall, M administration had no effect on ADG post-castration and only had minor impacts on behavior in calves band castrated pre-weaning.

## INTRODUCTION

Potentially painful procedures are a public concern because of interest in animal well-being by consumers (Broom, 2010). Extra label nonsteroidal anti-inflammatory drugs (NSAIDs) may present a means to ameliorate perceived negative effects of painful procedures on stress physiology and behavior of calves. Following surgical castration, peak plasma cortisol concentrations were lower in calves treated with the NSAID sodium salicylate i.v. (Coetzee et al., 2007). However, oral administration of aspirin (acetylsalicylic acid) failed to alter plasma cortisol concentrations (Coetzee et al., 2007). Treatment with the NSAID flunixin meglumine following surgical castration with epidural anesthesia resulted in a reduced indication of pain for 8 h post-castration (Currah et al., 2009). Administration of the NSAID carprofen in combination with epidural anesthesia prior to castration by external clamping of 5- to 6-month-old calves resulted in lower plasma cortisol concentrations than an epidural alone up to 48 h post-castration (Stilwell et al., 2008). Additionally, the NSAID carprofen administered via i.v. decreased plasma fibrinogen and haptoglobin concentrations in calves that were castrated using bloodless techniques (Pang et al., 2006). Meloxicam (M) is a promising NSAID for potential pain amelioration in cattle. Meloxicam can be administered orally with a long half-life in ruminants and pre-ruminants (Coetzee et al., 2009; Mosher et al., 2012). There have been multiple studies of the use of M at the time of castration in weaned calves (Coetzee et al., 2012; Repenning et al., 2013; Roberts et al., 2015; Olson et al., 2016; Marti et al, 2018; Meléndez et al., 2018b).

Despite the body of research conducted in weaned calves, few studies have been conducted that examine the effects of M use in pre-weaned calves and in calves after band castration. In studies of pre-weaned calves, Brown et al. (2015) observed improved gain for the first month after castration with M administration to calves surgically castrated near birth, but there was no effect of M administration on weaning weight. However, Meléndez et al. (2018a) found M administration reduced circulating concentrations of substance P, white blood cell counts, and number of tail flicks after surgical castration of 1-week-old calves, and Marti et al. (2018) observed reduced hair cortisol concentration by M administration to 1-week-old, band castrated calves. Buccal M administration prior to surgical castration reduced pain behavior and inflammation following surgical castration of 2- to 4-month-old calves (van der Saag et al., 2018). Thus, there is a gap in knowledge of the impact of M administration following band castration of older, pre-weaning age calves. In a comparison of band and surgical castrated 1-week, 2-month, and 4-month-old calves, Meléndez et al. (2017) observed the fewest behavioral changes and no physiological changes associated with acute pain in calves band castrated at 2 months of age compared to non-castrated calves. Furthermore, some producers prefer to allow calves to grow and allow more observation of phenotype before making the decision to castrate. The objective of this study was to determine the effects of oral M on growth performance, behavior, and indicators of inflammation of 3-month-old, pre-weaned beef calves following band castration.

## MATERIALS AND METHODS

### Animals, Housing, and Management

All animal procedures were approved by the Berry College Institutional Animal Care and Use Committee. Nursing purebred Angus bull calves from the cow-calf herd at Berry College (Rome, GA; Latitude = 34.302344773276765; Longitude = −85.19597053527832) were enrolled in this trial during spring time (March and April). Calves were allowed ad libitum nursing and maintained as a collective group on a 35-acre pasture of primarily fescue grass for the duration of the experiment. The calves were provided no supplemental feed. Water was freely available, provided by watering troughs and pond access.

### Experimental Design

During sample collection and treatment administration (d −14, 0, 14, and 28) calves were temporarily separated from their dams and restrained in a cattle chute. All calves were weighed (Digi-Star Stock Weigh 600 with 24″ load cells mounted to an aluminum platform) and scrotal circumferences were determined (Reliabull Scrotal Tape, Lane Manufacturing, Inc.) prior to castration (d −14). Six calves were selected to remain bulls based on pedigree and phenotype (BULL; age = 104.2 ± 6.1 d; BW = 172.1 ± 8.7 kg; scrotal circumference = 17.5 ± 0.4 cm). Remaining bull calves were assigned to band castrated (BAN; *n* = 8; age = 90.2 ± 6.5 d; BW = 146.3 ± 11.4 kg; scrotal circumference = 16.0 ± 0.5 cm) or band castrated with meloxicam (BAN + M; *n* = 9; age = 102.1 ± 6.2 d; BW = 146.0 ± 7.7 kg; scrotal circumference = 16.1 ± 0.3 cm). All castrations were performed using a Callicrate Bander (No-Bull Enterprises, St. Francis, KS). Groups were administered oral M or an empty porcine gelatin capsule (Torpac Veterinary Size Capsules, 10 mL capsule, Torpac by Custom Capsules Ltd.) using a balling gun. Meloxicam (ZyGenerics, Cadila Healthcare Ltd, NDC 68382-051-05) was administered by inserting a tablet into a porcine gelatin capsule prior to being administered to the animal using a balling gun at a dose of 2 mg/kg on d 0 and 14 of the experiment.

### Behavioral Assessment

On d 0, IceTags (IceRobotics Ltd, Edinburgh, Scotland) were placed on each animal. The IceTag is a three-axis accelerometer. Data capture can be stored for up to 60 days and give a detailed analysis of standing, lying, stepping, and the motion index of the animal. IceTags were placed on the left hind limb by loosely fitting the band around the distal metatarsal region of the limb, fastening the band on the medial aspect, and wrapping the limb and device with elastic bandage material in order to prevent rotation and slipping. After placement of the IceTag on the left hind limb, the calves were turned back onto the pasture with the adult cows. To reduce the risk of injury, the IceTag was rotated to the right hind limb on d 14 and fastened as described above. The IceTags were removed after the final sample collection on d 28.

### Growth Rate

Body weight was measured on d −14, 0, 14, and 28 as previously described to determine average daily gain (ADG).

### Fibrinogen and Haptoglobin Assays

On d 0, 14, and 28, 10 mL blood samples (K_2_EDTA reference 367841 and Serum reference 366431; BD Vacutainer Blood Collection Tubes) were collected from the coccygeal or jugular vein of each calf prior to the administration of any treatment. Blood samples were placed immediately on ice until plasma separation using centrifugation (3,000 × *g*; 10 min.) was completed approximately 2 to 3 h following collection. Plasma samples were stored at −80 °C until analysis. Plasma samples were analyzed for fibrinogen concentrations by the University of Tennessee Veterinary Medical Center Diagnostic Laboratory Service. Plasma fibrinogen concentrations were determined by heat precipitation using a refractometer as previously described (Millar et al., 1971). Serum samples were analyzed for haptoglobin concentrations by the Kansas State University Veterinary Diagnostic Laboratory. A semiautomated technique was used on separated serum samples as previously described for the determination of haptoglobin concentrations (Smith et al., 1998).

### Statistics

Behavior data were tested for effect of treatment, day, and interactions using procedures for repeated measures with JMP software (version 7, SAS Inst. Inc., Cary, NC). Fibrinogen and haptoglobin data were tested for effect of treatment, day (0, 14, and 28), and treatment × day interaction using procedures for repeated measures with JMP software. The ADG data were tested for effect of treatment, period (d −14 to 0, d 0 to 14, and d 14 to 28), and treatment × period interaction using procedures for repeated measures with JMP software. Means separation was performed using Student’s *t*-test when the main effect or interaction was significant (*P* < 0.05).

## RESULTS

**Figure 1.**
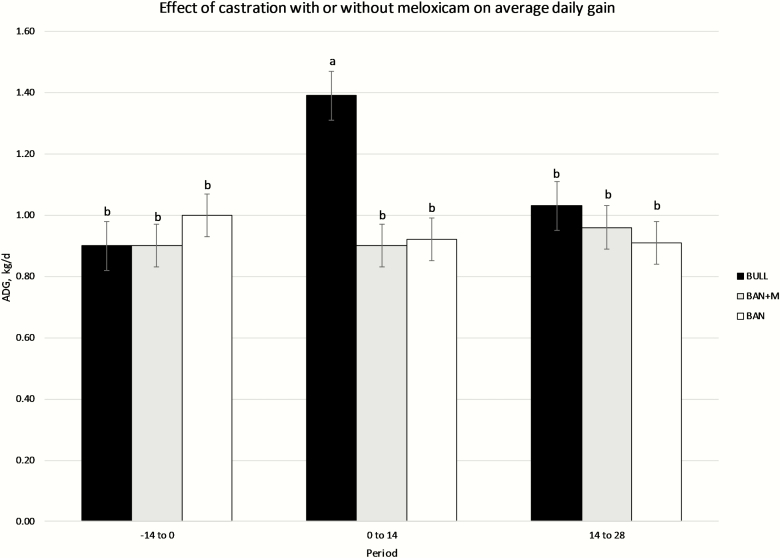
Average daily gain (LSMeans) of bulls selected based on pedigree and phenotype (BULL; *n* = 6), bulls castrated on d 0 using a Callicrate Bander (BAN; *n* = 8) and bulls castrated using a Callicrate Bander and administered meloxicam orally (2 mg/kg PO; BAN + M; *n* = 9) on d 0 and d 14. Effect of treatment, *P* = 0.0124; effect of period *P* = 0.0691, treatment × period, *P* = 0.0052. ^a,b^Bars that do not have a common letter differ (*P* < 0.05).

There was an effect of treatment (*P* = 0.0124) and treatment × period interaction (*P* = 0.0052) and a tendency for an effect of period (*P* = 0.0691) on ADG. Means separation indicated BULL in period d 0 to 14 had greater ADG than all other treatment period combinations, and BULL had greater ADG than BAN or BAN + M (Figure 1; *P* < 0.05).

**Figure 2.**
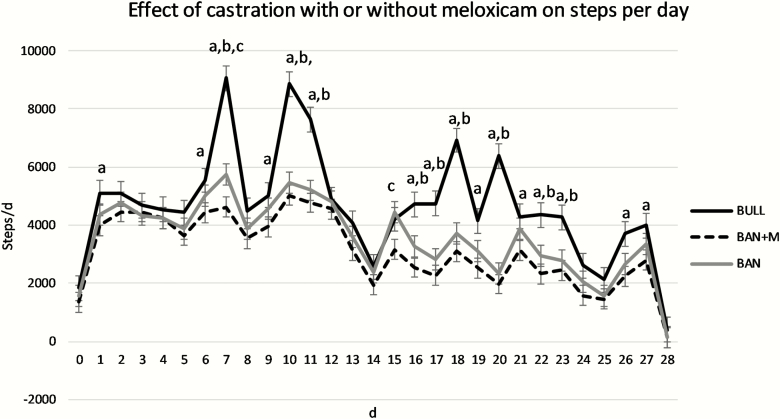
Steps per day (LSMeans) recorded by IceTags a three-axis accelerometers on bulls selected based on pedigree and phenotype (BULL; *n* = 6), bulls castrated on d 0 using a Callicrate Bander (BAN; *n* = 8) and bulls castrated using a Callicrate Bander and administered meloxicam orally (2 mg/kg PO; BAN + M; *n* = 9) on d 0 and d 14. IceTags were applied to the right rear leg on d 0, moved to the left rear leg on d 14, and removed on d 28. Effect of treatment, *P* = 0.0004; effect of day, *P* < 0.0001; treatment × day, *P* < 0.0001. ^a^Indicates BULL differs from BAN + M on the same day (*P* < 0.05). ^b^Indicates BULL differs from BAN on the same day (*P* < 0.05). ^c^Indicates BAN + M differs from BAN on the same day (*P* < 0.05).

For steps per day, there was an effect of treatment (*P* = 0.0004), day (*P* < 0.0001), and treatment × day interaction (*P* < 0.0001). BULL took more steps on d 1, 6, 7, 9, 10, 11, 16, 17, 18, 19, 20, 21, 22, 23, 26, and 27 than BAN + M. BULL also took more steps on d 7, 10, 11, 16, 17, 18, 20, 22, and 23 than BAN. On d 7 and 15, BAN took more steps than BAN + M. Overall, BULL took more steps per day than BAN or BAN + M (Figure 2; *P* < 0.05).

**Figure 3.**
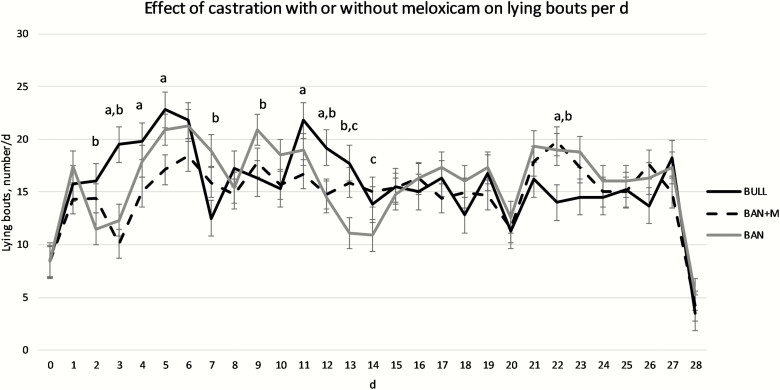
Number of lying bouts per day (LSMeans) recorded by IceTags a three-axis accelerometers on bulls selected based on pedigree and phenotype (BULL; *n* = 6), bulls castrated on d 0 using a Callicrate Bander (BAN; *n* = 8) and bulls castrated using a Callicrate Bander and administered meloxicam orally (2 mg/kg PO; BAN + M; *n* = 9) on d 0 and d 14. IceTags were applied to the right rear leg on d 0, moved to the left rear leg on d 14, and removed on d 28. Effect of treatment, *P* = 0.61; effect of day, *P* < 0.0001; treatment × day, *P* < 0.0001. ^a^Indicates BULL differs from BAN + M on the same day (*P* < 0.05). ^b^Indicates BULL differs from BAN on the same day (*P* < 0.05). ^c^Indicates BAN + M differs from BAN on the same day (*P* < 0.05).

There was no effect of treatment (*P* = 0.61), but there was an effect of day (*P* < 0.0001) and treatment × day interaction (*P* < 0.0001) on the number of lying bouts per day. BULL had more lying bouts on d 3, 4, 5, 11, and 12 than BAN + M. BULL also had more lying bouts on d 2, 3, 12, and 13 than BAN. BAN had more lying bouts than BULL on d 7, 9, and 22. BAN + M had more lying bouts than BULL on d 22. BAN + M had more lying bouts than BAN on d 13 and 14 (Figure 3; *P* < 0.05).

**Figure 4.**
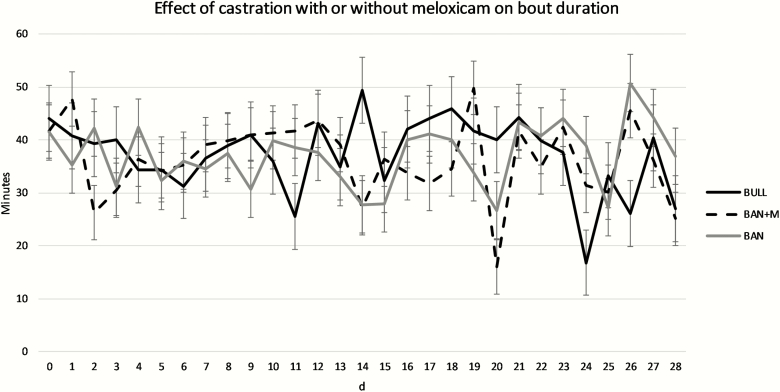
Lying bout durations (LSMeans) recorded by IceTags a three-axis accelerometers on bulls selected based on pedigree and phenotype (BULL; *n* = 6), bulls castrated on d 0 using a Callicrate Bander (BAN; *n* = 8) and bulls castrated using a Callicrate Bander and administered meloxicam orally (2 mg/kg PO; BAN + M; *n* = 9) on d 0 and d 14. IceTags were applied to the right rear leg on d 0, moved to the left rear leg on d 14, and removed on d 28. Effect of treatment, *P* = 0.76; effect of day, *P* = 0.0095; treatment × day, *P* = 0.072.

For lying bout duration, there was no effect of treatment (*P* = 0.76) or treatment × day interaction (*P* = 0.072), but there was an effect of day (Figure 4; *P* = 0.0095).

**Figure 5.**
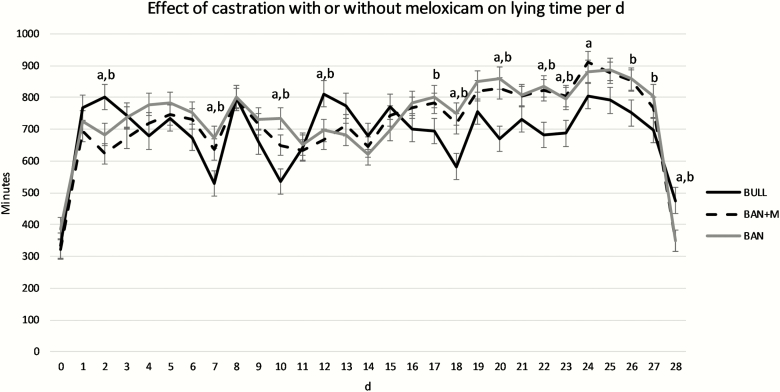
Lying time per day (LSMeans) recorded by IceTags a three-axis accelerometers on bulls selected based on pedigree and phenotype (BULL; *n* = 6), bulls castrated on d 0 using a Callicrate Bander (BAN; *n* = 8) and bulls castrated using a Callicrate Bander and administered meloxicam orally (2 mg/kg PO; BAN + M; *n* = 9) on d 0 and d 14. IceTags were applied to the right rear leg on d 0, moved to the left rear leg on d 14, and removed on d 28. Effect of treatment, *P* = 0.367; effect of day, *P* < 0.0001; treatment × day, *P* < 0.0001. ^a^Indicates BULL differs from BAN + M on the same day (*P* < 0.05). ^b^Indicates BULL differs from BAN on the same day (*P* < 0.05).

For lying time per day, there was no effect of treatment (*P* = 0.367), but there was an effect of day (*P* < 0.0001) and treatment × day interaction (*P* < 0.0001). BULL spent more time per day lying on d 2, 12, and 28 than BAN + M and BAN. BAN + M and BAN spent more time per day lying on d 7, 10, 18, 20, 22, and 23 than BULL. BAN also spent more time per day lying on d 17, 26, and 27 than BULL. BAN + M spent more time per day lying on d 24 than BULL (Figure 5; *P* < 0.05).

**Figure 6.**
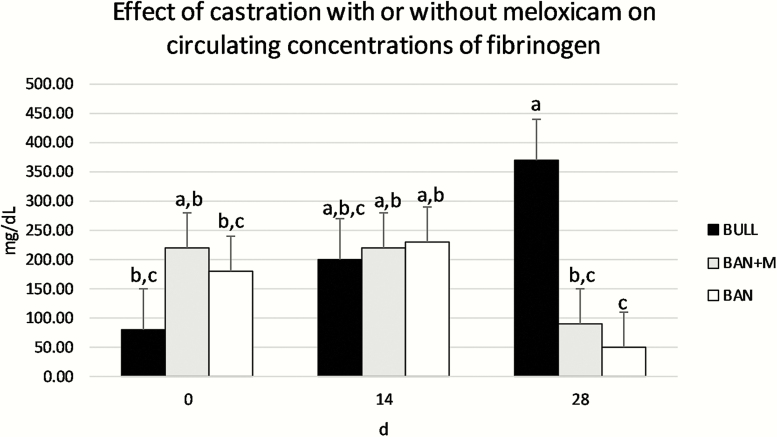
Plasma concentrations of fibrinogen (LSMeans) of bulls selected based on pedigree and phenotype (BULL; *n* = 6), bulls castrated on d 0 using a Callicrate Bander (BAN; *n* = 8) and bulls castrated using a Callicrate Bander and administered meloxicam orally (2 mg/kg PO; BAN + M; *n* = 9) on d 0 and d 14. Effect of treatment, *P* = 0.46; effect of day, *P* = 0.51; treatment × day, *P* = 0.014. ^a,b,c^Bars that do not have a common letter differ (*P* < 0.05).

**Figure 7.**
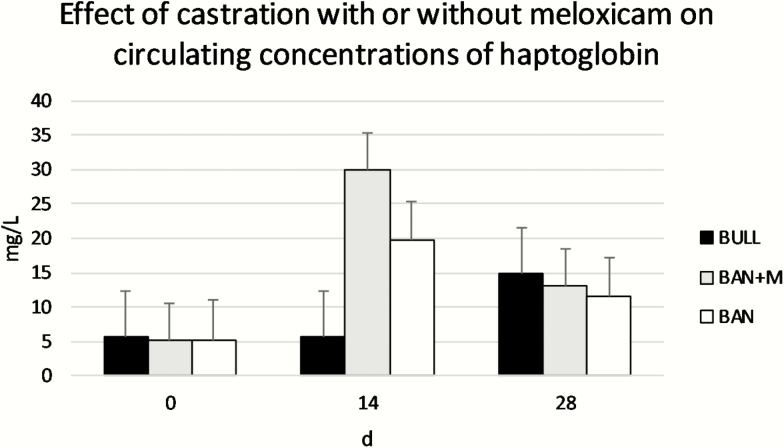
Serum concentrations of haptoglobin (LSMeans) of bulls selected based on pedigree and phenotype (BULL; *n* = 6), bulls castrated on d 0 using a Callicrate Bander (BAN; *n* = 8) and bulls castrated using a Callicrate Bander and administered meloxicam orally (2 mg/kg PO; BAN + M; *n* = 9) on d 0 and d 14. Effect of treatment, *P* = 0.52; effect of day, *P* = 0.0085; treatment × day, *P* = 0.086. Serum concentrations of haptoglobin were greater on d 14 than d 0, but serum concentrations of haptoglobin on d 28 did not differ from d 0 or 14.

For plasma concentrations of fibrinogen (Figure 6), there was no effect of treatment (*P* = 0.46) or day (*P* = 0.51), but there was a treatment × day interaction (*P* = 0.014). BULL had greater circulating concentrations of fibrinogen on d 28 than BULL on d 0, BAN + M on d 28, and BAN on d 28, and BAN had the lower circulating concentration of fibrinogen on d 28 than BAN or BAN + M on d 14 or BAN + M on d 0 (*P* < 0.05). For plasma concentrations of haptoglobin (Figure 7), there was a tendency for a treatment × day interaction (*P* = 0.086) such that plasma concentrations of haptoglobin for BAN + M on d 14 were greater than BAN + M on d 0 or 28, BAN on d 0 or 28, and BULL on d 0 and 14. Plasma concentrations of haptoglobin for BAN on d 14 were also greater than BAN on d 0. There was no effect of treatment (*P* = 0.52) but there was an effect of day (*P* = 0.0085) such that plasma concentrations of haptoglobin were greater on d 14 than d 0 but plasma concentrations of haptoglobin on d 28 did not differ from d 0 or 14.

## DISCUSSION

In the current study, the administration of M did not impact ADG of the band castrated bulls in the 28 days following castration. Likewise, the administration of a buccal transmucosal formulation of M prior to surgical castration in 2- to 4-month-old calves had no effect on ADG to 6 days post-castration (van der Saag et al., 2018). Similarly, subcutaneous M administration immediately prior to band or surgical castration did not affect ADG to 56 days post-castration in 7- to 8-day-old calves (Marti et al., 2018). Subcutaneous M administration 30 min prior to surgical castration of 6- to 8-month-old *Bos indicus* calves under general anesthesia had no effect on ADG to 13 days post-castration (Musk et al., 2017). Additionally, subcutaneous M administration 30 min prior to surgical or band castration had no effect on ADG to 28 days post-castration in 7- to 8-month-old calves (Meléndez et al., 2018b). There was also no effect of oral M administration 24 h prior to surgical castration on ADG of 8- to 10-month-old beef calves (Coetzee et al., 2012), and Repenning et al. (2013) observed no effect on ADG of oral M administration in band castrated yearling beef bulls. Furthermore, intravenous administration of carprofen 20 min prior to castration using either a band or burdizzo had no effect on ADG to 35 days post-castration (Pang et al., 2006). However, the combination of administration of local anesthesia and intravenous ketoprofen prior to castration resulted in increased ADG to 35 days post-castration (Earley and Crowe, 2002). If there is an impact of NSAID administration alone at castration on ADG, it is likely insignificant to overall growth performance.

Interestingly, bulls that were not castrated only had an advantage in ADG in the 14-day period immediately following the application of the band. Others have also demonstrated no difference in pre-weaning growth between bulls that are castrated at less than 3 months of age and bulls that remain intact until weaning (Lents et al., 2006; Brown et al., 2015). Marti et al. (2017) reported calves surgically castrated at 1 week of age had lower ADG to weaning than intact bulls and calves band castrated at 1 week of age, but they did not observe any difference in ADG to weaning between intact bulls and calves surgically or band castrated at 2 or 4 months of age.

On d 15 in the current study, one day following administration of the second dose of M, BAN + M took fewer steps than BAN. BAN + M also took fewer steps on d 7 than BAN. Repenning et al. (2013) found no impact of oral M administration to band castrated yearling bulls on behavior. Interestingly, on d 7, 10, 11, 16, 17, 18, 20, 22, and 23 bulls took more steps than either group of banded calves. It is possible these were days that there were cows in the pasture in estrus. Unfortunately, this study did not include estrous detection, and we can therefore not test this hypothesis. On d 13 and 14, BAN + M had more lying bouts per day than BAN. There were also many days on which bulls had more lying bouts. Meléndez et al. (2018b) observed reduced physiological indicators of pain but no effect on behavioral indicators in surgically castrated, 7- to 8-month-old calves treated with M. Van der Saag et al. (2018) observed a reduction in the frequency of foot stamps and duration of time spent walking with a stiff hypometric gate with administration of buccal transmucosal M gel prior to surgical castration in 2- to 4-month-old calves. Subcutaneous M administration at the time of surgical castration in 7- to 8-day-old calves increased the number of tail flicks observed (Marti et al., 2018). Olson et al. (2016) observed a reduction time spent lying and number of lying bouts as well as an increase in motion index and number of steps for 3 days following castration with oral administration of M 2 h prior to band or surgical castration of 4- to 5-month-old Holstein calves. Administration of M subcutaneously at the time of castration reduced circulating concentrations of substance P and the number of tail flicks in week-old calves castrated by either banding or surgically (Meléndez et al., 2018a). Meléndez et al. (2017) also observed the fewest behavioral and no physiological changes associated with acute pain in band castrated 2-month-old calves compared to non-castrated calves in a study of surgical versus band castration of 1-week, 2-month, and 4-month-old calves. Furthermore, Marti et al. (2017) reported on the same study that there was no effect of castration method on behavioral indicators of chronic pain in 1-week or 2-month-old calves. Thus, the timing and method of castration in this study were less likely to result in behavioral indicators of pain. Although M administration only reduced steps per day on d 7 and 15 and increased lying bouts per day on d 13 and 14 in the current study, other studies have indicated M administration may ameliorate the pain associated with castration.

Both fibrinogen and haptoglobin are acute phase indicators of inflammation (Petersen et al., 2004). Neither fibrinogen nor haptoglobin concentrations in response to castration were altered by M administration in the current study. Musk et al. (2017) also failed to observe any effect of M administration on circulating concentrations of haptoglobin or fibrinogen following surgical castration of 6- to 8-month-old *Bos indicus* calves. Additionally, circulating concentrations of haptoglobin and fibrinogen were not affected by the subcutaneous administration of M at the time of band or surgical castration in 7- to 8-day-old calves (Marti et al., 2018). However, others have observed altered inflammatory indicators shortly following castration with the administration of M and other NSAIDs. Brown et al. (2015) observed attenuated the haptoglobin response to surgical castration in calves post-weaning on d 3 post-castration with oral M administration, but haptoglobin concentrations had returned to baseline levels by 7 days post-castration. Additionally, Roberts et al. (2015) observed a reduction in serum haptoglobin concentrations at 36 and 60 h after surgical castration with oral meloxicam administration. Pang et al. (2006) observed lower plasma concentrations of fibrinogen on d 14 and lower plasma concentrations of haptoglobin in band castrated calves treated with carprofen prior to band application than band castrated calves. Subcutaneous M administration 30 min prior to surgical castration reduced circulating concentrations of haptoglobin 24 and 48 h after castration but had no effect at 14 days after castration (Meléndez et al., 2018b). Similarly, intravenous administration of ketoprofen prior to castration reduced the increase in haptoglobin on d 1 following surgical castration and ketoprofen in combination with local anesthetic reduced the increase in fibrinogen immediately following castration (Earley and Crowe, 2002). However, the effects of ketoprofen on haptoglobin and fibrinogen were not detectable by 7 days after castration (Earley and Crowe, 2002). Although band castration wounds are not resolved by 14 days post-castration (Marti et al., 2017), it is likely the timing of blood collection in the current study precluded the observation of an effect of M administration of circulating concentrations of fibrinogen or haptoglobin. Bulls that were not castrated had greater circulating concentrations of fibrinogen on d 28. However, the concentrations of fibrinogen observed in all animals in the study were low and generally what would be expected in healthy animals (250 to 800 mg/dL, McSherry et al., 1970; Petersen et al., 2004).

In conclusion, in the current study, oral M administration at the time of castration and 14 days after castration did not result in improved animal performance or major improvements in the behavioral indicators of pain measured. The method of castration and age of the calves in the current study may have contributed to the lack of an impact of oral M administration. However, it is also important to note oral M administration at the time of castration and 14 days after castration did not result in any observed negative impacts and others have observed M to ameliorate indicators of pain.
